# Enhancing productivity, modifying biochemical parameters, and regulating the phenylpropanoid pathway in 'Le-Conte' pears through optimal protocatechuic acid treatments

**DOI:** 10.1186/s12870-023-04715-9

**Published:** 2024-01-15

**Authors:** Emad Hamdy Khedr, Nagwa Khedr

**Affiliations:** Department of Pomology, Faculty of Agriculture, Cairo University, Giza, 12613 Egypt

**Keywords:** Antioxidant, Fruit quality, *Pyrus communis*, Pre-harvest, Yield, Sustainable agriculture

## Abstract

**Background:**

This study aimed to investigate the impact of protocatechuic acid (PRC) treatments on the productivity and fruit quality of 'Le-Conte' pears, with a specific focus on productivity, stone cells content, and antioxidant activity. The research spanned over three consecutive cultivating seasons, with the first season serving as a preliminary study to determine the optimal PRC concentrations and the most effective number of spray applications. During the initial season, response surface methodology (RSM) was employed to optimize PRC concentration and application frequency. PRC was evaluated at concentrations ranging from 50 to 400 ppm, with treatment frequencies of either once or twice. Considering the optimal conditions obtained from RSM results, PRC treatments at 200 ppm and 300 ppm were applied twice, and their respective effects were studied in comparison to the control in the following seasons.

**Results:**

RSM results indicated that PRC at 200 and 300 ppm, applied twice, once during full bloom and again three weeks later, yielded the most significant effects. Subsequent studies revealed that PRC treatments had a substantial impact on various aspects of fruit production and quality. Applying 300 ppm PRC once during full bloom and again three weeks later resulted in higher fruit set percentages, lower fruit abscission, and enhanced fruit yield compared to untreated trees. Additionally, the 200 ppm PRC treatment maintained physicochemical characteristics such as fruit color, increased total soluble solids (TSS), and total sugar, and maintained higher ascorbic acid content and antioxidant capacity in the fruits while reducing stone cells content and lignin. Notably, enzyme activities related to phenylpropanoid metabolism and stone cells, including phenylalanine ammonia-lyase (PAL), cinnamate-4-hydroxylase (C4H), 4-Coumarate-CoA Ligase (4CL), cinnamyl alcohol dehydrogenase (CAD), and cinnamoyl-CoA reductase (CCR), as well as peroxidase, polyphenol oxidase, and laccase, were significantly regulated by PRC treatments.

**Conclusion:**

Overall, this study suggests that PRC treatments are suitable for enhancing pear yield and quality, with PRC at 200 ppm being the more recommended option over 300 ppm. This approach serves as an effective strategy for achieving a balance between enhancing the productivity and fruit quality of 'Le-Conte' pears.

**Supplementary Information:**

The online version contains supplementary material available at 10.1186/s12870-023-04715-9.

## Introduction

Pears (*Pyrus spp.*) are deciduous fruit trees that belonging to the Rosaceae family. The 'Le-Conte' pear, a widely cultivated cultivar in Egypt and many other countries is an interspecific hybrid resulting from a cross between *Pyrus communis* and *Pyrus pyrifolia* [1]. Satisfying market demands through the increase of pear productivity and the enhancement of fruit quality are central objectives in pear production. Several factors influence the productivity of pear trees, and low average production is often attributed to partial self-incompatibility, resulting in increased abscission and reduced yield [2], in addition to nutrient deficiency, and other significant factors including susceptibility to various diseases [3]. Also, pear fruits are known for their distinctive textures, which can vary among different pear varieties. Some pear varieties are characterized by a gritty texture, primarily due to the presence of stone cells [4]. These stone cells have thick cell membranes and limited internal space, which can contribute to the slow ripening of pears [5], in addition to higher oxidative stress in fruits [6].

In light of these characteristics, the pre-harvest application of antioxidant agents has emerged as a promising strategy to address these issues and improve the overall quality of pear fruits [7]. Antioxidants play a crucial role in the defense mechanisms of plants against oxidative stress caused by reactive oxygen species (ROS) [8]. The accumulation of ROS, which can result from normal metabolic processes or environmental stresses, can lead to cellular damage, and have a detrimental impact on fruit development, quality, and post-harvest storability [9]. The application of antioxidants or antioxidant-enhancing compounds via sprays is hypothesized to reduce oxidative stress in pear trees, thereby enhancing both fruit yield and quality [5, 6]. Numerous studies have explored the effects of foliar antioxidant treatments on various fruit crops, including pears. For instance, Medan et al. [10] investigated the impact of foliar sprays containing ascorbic acid on the quality and yield of pears. Their findings indicated increased antioxidant activity, reduced disorders related to oxidative stress, improved fruit quality, and prolonged storage. Similarly, Zargar et al. [11] conducted a study involving the application of different compounds as pre-harvest sprays on pears, noting improvements in fruit color, firmness, and overall quality attributes.

Stone cells, which are commonly found in most pear cultivars, play a significant role in determining the internal quality of pear fruit [12]. These cells affect not only the sucrose content but also the flesh hardness, adhesiveness, and chewiness [13]. The formation of stone cells originates from the lignification of parenchyma cells, forming what is known as the stone cells primordium [14]. These primordia become visible in the flesh approximately 15 days after full bloom (DAFB) and develop into clusters of stone cells by around 60 DAFB, persisting in varied ranges at maturity [15]. Lignin, which constitutes 20-30% of stone cells, is a key contributor to both cell wall thickening and stone cells formation [16].

The phenylpropanoid pathway is important in plants because it produces a wide range of metabolites [17]. It is required for the synthesis of lignin and serves as a precursor for various important metabolites such as lignin, flavonoids, and coumarins. A range of phenolic polymers and lignin are generated within the phenylpropanoid pathway [18], contributing to numerous disease resistance mechanisms in plants [19]. Furthermore, the intermediate phenylpropanoid compounds produced during lignin production have antibacterial characteristics and play an active role in plant defense.

Enhancing the productivity and quality of pear trees is a primary goal for growers. With the increasing demand for sustainable agricultural practices, the exploration of natural compounds with potential plant health benefits has gained significant attention [1]. One such compound is protocatechuic acid (PRC). PRC is a natural acid widely distributed in plants and is known for its notable antioxidant and bioactive properties [20]. PRC is generally considered safe for human consumption. It is a dietary polyphenol with potential health benefits, and it has been the subject of research for its various pharmaceutical and medicinal applications [20]. Different studies have highlighted its potential role in promoting plant growth [21], mitigating abiotic stresses, and improving fruit quality [22]. By applying PRC as a foliar treatment, it is hypothesized that its beneficial effects on pear trees may include enhanced nutrient uptake, increased photosynthetic efficiency, and improved resistance to pathogens and environmental stresses [22].

The principal aim of this study is to comprehensively explore the impacts of pre-harvest sprays employing PRC as a potent antioxidant agent on pear trees. This investigation is geared towards striking a harmonious equilibrium between crop yield and the inherent attributes of fruit quality. The central focus lies in augmenting productivity and elevating the overall fruit quality, with a particular emphasis on mitigating stone cells formation during the critical phases of fruit development.

## Materials and methods

### Plant Material

The study focused on 'Le-Conte' pear (*Pyrus communis*) trees that were seventeen years old and grafted onto 'Communis' pear rootstock. These trees were planted in sandy soil, with a distance of 5x5 meters between each planting. A drip irrigation system was used (Tables S1 and S2). The investigation took place in Beheira Governorate, Egypt (30°17'34.3"N 30°31'42.8"E). The trees demonstrated consistent and uniform growth, showcasing optimal vigor. They underwent recommended fertilization and adhered to prescribed cultural practices. These conditions were consistently maintained throughout the research period, which spanned from 2020 to 2022. All fruits used in our experiments were purchased from a private field with the landowner's permission.

### Experimental design and treatments

The research extended across three consecutive cultivating seasons, with the initial season serving as a preliminary study to identify the optimal PRC concentrations and the most effective number of spray applications. In the initial season, response surface methodology (RSM) was utilized to optimize both PRC concentration and application frequency. In this study, PRC was evaluated at concentrations ranging from 50 to 400 ppm, with treatment frequencies of either once or twice (at the full bloom stage, at 3 weeks post full bloom, or at 6 weeks post full bloom). The study involved a total of sixty-five trees. The goal was to identify the optimal concentrations and application frequencies for the treatments throughout the season. To achieve this, a central composite design (CCD) approach was utilized. This process included developing a matrix of treatments that encompassed a range of concentrations and application frequencies. The design included both low and high levels of independent variables, and replicated center points were incorporated to assess experimental error.

This design applied a two-factor mixed-level experimental design and RSM. The analysis of data and the construction of the response surface methodology were carried out using Design Expert software, version 11 (Stat-Ease Inc., Minneapolis, MN, USA). The CCD consisted of 13 experimental runs, with five replicates at the center point. The measured responses included initial fruit set, fruit yield, total phenolic content, and antioxidant capacity. The relationship between the dependent variables and independent variables was represented by the following equation:

$${\text{Z}}= {{\text{C}}}_{0} +\Sigma {{\text{C}}}_{i} {{\text{X}}}_{i} +\Sigma {{\text{C}}}_{ii} {{{\text{X}}}_{ii}}^{2} +\mathrm{ \Sigma \Sigma }{{\text{C}}}_{ij} {{\text{X}}}_{i} {{\text{X}}}_{j}$$

The responses were signified as Z, with the intercept signified by C_0_. The regression coefficients for the linear, quadratic, and interaction effect relations were represented as C_i_, C_ii_, and C_ij_, respectively. The independent variables were represented as X_i_ and X_j_. Various analyses were conducted to determine the optimal conditions for PRC concentration and treatment time in relation to the productivity and quality of the 'Le-Conte' pear, including analysis of variance, regression, and surface plotting.

RSM results showed that PRC at 200 and 300 ppm, applied twice, yielded the most significant effects. A total of 45 trees were selected in the advanced experiments conducted during the 2021 and 2022 seasons. Three treatments, based on the results from RSM, were applied, each replicated five times, with three trees in each replicate. The treatments included the application of 200 ppm PRC, 300 ppm PRC, and a control group that was sprayed with water only. The treatments were applied by spraying the respective solutions, enhanced with the addition of a surfactant (Tween 20), until runoff on mature pear trees, using a manual pump sprayer. PRC foliar treatments were conducted twice, once during the full bloom stage (at the point when 70% of the flower buds had fully opened) and similarly, three weeks after reaching the full bloom stage. The timing of the treatments adhered to the recommended guidelines, considering weather conditions, and avoiding periods of elevated temperature, vigorous winds, or rainfall.

### Effect of various PRC treatments on the productivity of 'Le-Conte' pear trees

In every season of the study (2020, 2021, and 2022), five shoots were chosen randomly on each pear tree, and these shoots were marked at the start of the growing season. The objective was to guarantee a representative sample from various sides of the tree. The count of inflorescences on each marked shoot was recorded, and a random sample of thirty inflorescences was selected to calculate the average number of flowers per inflorescence. This approach was designed to acquire a dependable estimate of the average number of flowers. Three weeks after reaching the full bloom stage, the initial fruit set percentage was calculated using the following formula: initial fruit set percentage = [(total number of fruits per shoot) / (average number of flowers per inflorescence × number of inflorescences per shoot)] × 100 [1].

Furthermore, the percentage of fruit abscission was determined by calculating the proportion of fruits that underwent abscission at harvest. This calculation involved dividing the number of abscised fruits at harvest by the total number of fruit sets and then multiplying by one hundred. To obtain the number of abscised fruits, the total number of fruits at harvest was subtracted from the total number of fruit set on the marked branches for each tree. This analysis helped evaluate the extent of fruit abscission during the study. At the harvest stage, which occurred 130 days after the full bloom, the fruit yield in Kg was recorded for each tree included in the study.

### Effect of different PRC treatments on physicochemical characteristics of pear fruits

During the harvest period, a random selection of forty-five fruits from each treatment was sampled. This comprised nine fruits from each replicate. The sampled fruits were used to determine various fruit characteristics, including average fruit weight (g), shape index (length-to-diameter, L/D ratio), and specific gravity. The specific gravity was calculated by determining the fruit weight and volume (in grams per cubic centimeter). Fruit firmness was assessed through an instrumental test conducted with a force-torque tester (Mecmesin, England) equipped with an 8 mm diameter probe [23]. Measurements were taken on multiple points on the surface of each pear fruit, and the data were presented in Newtons (N).

Color measurements were conducted on distinct areas of both the peel and flesh surfaces of the pear fruit. The measurements were objectively carried out using a Minolta colorimeter (Model CR-400, Minolta, Osaka, Japan) based on the CIE L* a* b* values [24]. The L* value signified the brightness of the color, with higher values indicating increased brightness. Meanwhile, the a* value represented chromaticity on the green (negative values) to red (positive values) axis. Total soluble solids (TSS) were ascertained by placing drops of pear fruit juice on a digital refractometer (PAL-1, ATA-GO Co., Ltd., Tokyo, Japan).

To evaluate the total sugar content, the method outlined by Nielsen [25] was applied. A sample comprising 5 g of fruit flesh was extracted, and the total sugar content was determined using a colorimetric method involving a reaction with H_2_S0_4_. The result of this analysis was expressed as grams of sugar per 100 grams of fresh fruit flesh.

To determine the ascorbic acid content, a titration method was utilized, as described by Khedr [26]. The analysis involved titrating the fruit extract against a dye solution containing 2,6-dichlorophenol-indophenol. The results were expressed as mg of ascorbic acid per 100 g of fresh fruit weight (FW).

### Determination of lignin and stone cells

Samples of 5 fruits were randomly selected from each replicate tree for every treatment, and these samples were collected at five different time points; 60, 70, 80, 90, and 100 DAFB. The evaluation of lignin content in the fruit was conducted using the thioglycolate lignin method, following the procedures outlined by Cai et al. [27]. Quantification of stone cells was performed based on the method detailed by Lu et al. [14]. Approximately 15g of the fruit sample underwent homogenization and subsequent dilution with a 0.1 M NaCl solution. The resultant suspension was then subjected to incubation at 22°C for 30 min. Following this, the sediment obtained underwent another 30 min incubation, this time with 0.25 L of 0.5 M NaOH. Subsequently, the sediment was suspended in 0.25 L of 0.5 M HCl for an additional 30 min and was then thoroughly washed with distilled water. This washing process was repeated several times to ensure the whole removal of any extraneous cell debris from the stone cells.

### Determination of total phenolic content (TPC), and total antioxidant capacity (TAC)

The assessments were conducted at five time points after full bloom, specifically at 60, 70, 80, 90, and 100 days, to determine both the TPC and TAC of the samples. The determination of TPC was conducted using the Folin-Denis reaction method, as described by Waterhouse [28], with measurements taken at a wavelength of 765 nm. The quantification was performed by referencing a standard curve with known gallic acid concentrations, and the outcomes were reported in mg of gallic acid per 100 g of fresh weight. For the evaluation of TAC, the ability to scavenge free radicals was assessed at 515 nm, following the methodology outlined by Dragović-Uzelac et al. [29].

### Measurement of antioxidants and quality- related enzymes

The determination of cinnamate 4-hydroxylase (C4H, EC 1.14.13.11) activity followed the procedure outlined by Liu et al. [30]. To initiate the process, 1 g of fruit tissue was homogenized in 3 mL of 50 mM Tris-HCl buffer with a pH of 8.7. Subsequently, the homogenate was subjected to centrifugation at 12,000 g for 20 min at a temperature of 4°C. The resulting supernatant was collected for the assessment of enzyme activity, with C4H activity being monitored at 340 nm.

The measurements of 4-Coumarate-CoA Ligase (4CL; EC 6.2.1.12) and cinnamyl alcohol dehydrogenase (CAD, EC 1.1.1.195) activities at the harvest stage were carried out following the procedure outlined by Takshak and Agrawal [31]. Initially, 1 g of the sample was ground with 2 mL of ice-cooled 0.2 M Tris-HCl buffer at pH 7.9, which also contained 2% PVP, 0.1% Triton X-100, 8 mM MgCl_2_, 1 mM PMSF, and 5 mM dithiothreitol. Subsequently, the resulting mixture was subjected to centrifugation at 12,000 g for 20 min at 4°C, and the supernatant was collected for enzyme extraction, the activity of 4CL was monitored at 333 nm. CAD was assayed spectrophotometrically, and the rate of consumption of NADPH in the presence of coniferaldehyde was monitored spectrophotometrically at 340 nm.

The determination of phenylalanine ammonia-lyase (PAL, E.C. 4.1.3.5) activity followed the method outlined by Han et al. [32]. Initially, 1 g of fruit flesh was homogenized with 2 mL of 50 mM sodium borate buffer at pH 8.7. PAL enzyme activity was quantified in units (U), with each unit representing the amount of PAL that resulted in an increase in absorbance at 290 nm of 0.01 per minute. Cinnamoyl-CoA reductase (CCR, E.C. 1.2.1.44) activity was assessed using the technique described by Sonawane et al. [33]. The measured CCR activity was determined at 366 nm. Laccase (EC 1.10.3.2) activity was evaluated using a modified method based on Bourbonnais and Paice method [34]. Initially, 1 g of flesh was homogenized with 2 mL of ice-cold 0.2 M NaAc-HAc buffer at pH 4.4. The mixture was then subjected to centrifugation at 12,000 g for 20 min at 4°C to initiate the reaction. The reaction was conducted at 25°C, and the change in absorbance at 420 nm was measured over a period of 5 min.

The peroxidase (POD, EC 1.11.1.7) activity was determined using a method based on Zheng et al. [35]. The reaction mixture consisted of 2.7 mL of 0.03% H_2_O_2_ in 100 mM sodium phosphate buffer at pH 6.2, along with 0.2 mL of the POD extract sample. The enzymatic reaction was initiated by adding 0.1 mL of a 1% (w/v) o-Dianisidine solution in methanol. The initial alteration in absorbance was measured at 460 nm. Polyphenol oxidases (PPO, E.C. 1.14.18.1) activity was measured using a modified spectrophotometric method [36]. The reaction mixture included 0.5 mL of the extract, 0.8 mL of 100 mM sodium phosphate buffer at pH 7.5, and 0.05 mL of 10 mM catechol solution. This mixture was then incubated for 30 min at 30°C. After incubation, 0.8 mL of a 2 M perchloric acid solution was added, and the tubes were placed in an ice bath. The absorbance was recorded at 420 nm.

### Statistical analysis

To determine the most effective concentration and application frequency of PRC to maximize productivity and enhance fruit quality. RSM data were investigated with Design Expert software (version 11.0, Stat-Ease Inc., Minneapolis, MN, USA). The influence of PRC concentration and treatment time on several responses (initial fruit set, fruit yield, TPC, and TAC) was evaluated through ANOVA, examining the linear, quadratic, and interaction effects of the independent variables. For statistical significance, *P*-values of less than 0.05 were considered. The physicochemical and fruit quality attributes of the pear were analyzed using MSTAT-C software (Michigan State University, USA). The experimental design followed a completely randomized block design. Results are presented as the means ± standard error (SE). The Post hoc Duncan test was applied with a significance level of 0.05.

## Results

### Model fitting and optimizing PRC treatments

In this investigation, we examined the impacts of PRC treatments on the initial fruit set, fruit yield, TPC, and TAC of pear. Table 1 displays the ANOVA results for model validation and adequacy. The R^2^ values, ranged from 0.592 to 0.820 for the initial fruit set, fruit yield, TPC, and TAC. These values suggest that over 59% of the total variation in the traits was accounted. The created models displayed varying degrees of significance for the assessed parameter, with significance observed for all measured responses. It is noteworthy that robust statistical models are characterized by comparable values of R^2^, adjusted R^2^, and predicted R^2^ [37].

**Table 1:** The regression coefficients for the process variables and corresponding product responses

**Factors**	**Initial fruit set**	**Fruit yield**	**Total phenolic content**	**Antioxidant capacity**
*Intercept*				
β_0_	15.82	87.46	34.92	37.10
*Linear*				
X_1_ (β_1_)	2.26	7.07	0.383	0.588
X_2_ (β_2_)	1.96	13.29	0.897	0.461
*Interaction*				
X_1_X_2_ (β_12_)	1.09	-0.942	0.344	0.164
*Quadratic*				
X_1_^2^ (β_11_)	-2.75	-11.97	-1.12	-0.91
X_2_^2^ (β_22_)	-0.97	-1.94	-0.697	-0.23
Model F-value	6.59	3.80	2.03	1.54
*P*-value	0.0140	0.047	0.039	0.047
Mean	14.36	82.15	34.13	36.64
C.V. %	11.60	12.38	7.40	8.23
Adeq. precision	7.44	6.73	5.43	4.20
R^2^	0.820	0.730	0.592	0.779
Adjusted R^2^	0.702	0.631	0.601	0.857
Std. Dev.	1.67	10.17	1.16	1.55

The adjusted R^2^ values for the initial fruit set, fruit yield, TPC, and TAC in this investigation varied from 0.601 to 0.857. Moreover, all parameters demonstrated a strong correlation between the predicted and actual values. Another factor indicating the suitability of the model is adequacy precision. A high adequacy precision (more than 4) is considered desirable [38]. In our study, the adequacy values ranged from 4.20 to 7.44. In this study, the CV values for the initial fruit set, fruit yield, TPC, and TAC ranged from 7.40 to 12.38. These values suggest high precision and reproducibility of the experimental data, along with a good fit of the used models. The results of this study demonstrate that the experimental data were reliable, and adequate for optimizing PRC treatment to enhance the productivity, total phenolic content, and total antioxidant capacity of the 'Le-Conte' pear.

The impact of the treatment variables (PRC concentration and PRC treatment repetition) on the initial fruit set, fruit yield, TPC, and TAC of pear was noted to fluctuate (Table 1.). Increasing the concentration of PRC had a positive effect on all the measured responses, suggesting that higher PRC concentration may enhance productivity and TAC. Moreover, the interaction among PRC concentration, treatment repetition, and time exhibited varied effects on the tested responses. To describe the influence of significant factors such as PRC concentration and treatment time on the responses (initial fruit set, fruit yield, TPC, and TAC) of pear, the following equation was derived:

$$\begin{array}{l}{{\text{Z}}}_{\text{Initial fruit set}}= 5.35 - 0.0440\text{A} - 1.73\text{B} - 0.0011\text{AB} + 0.243\text{A}^{2} - 0.0032\text{B}^{2}\\ \text{Z}_{\text{Fruit yield}}= 32.5 - 0.2240\text{A} - 10.20\text{B} - 0.0041\text{AB} + 0.490\text{A}^{2} - 0.0034\text{B}^{2}\\ \text{Z}_{\text{Total phenolic content}}= 30.34 - 0.0156\text{A} - 1.27\text{B} - 0.0043\text{AB} + 0.174\text{A}^{2} - 0.0019\text{B}^{2}\\ \text{Z}_{\text{Antioxidant capacity}}= 33.92 - 0.0154\text{A} - 0.48\text{B} - 0.0039\text{AB} + 0.060\text{A}^{2} - 0.0047\text{B}^{2}\end{array}$$

**Fig. 1:**
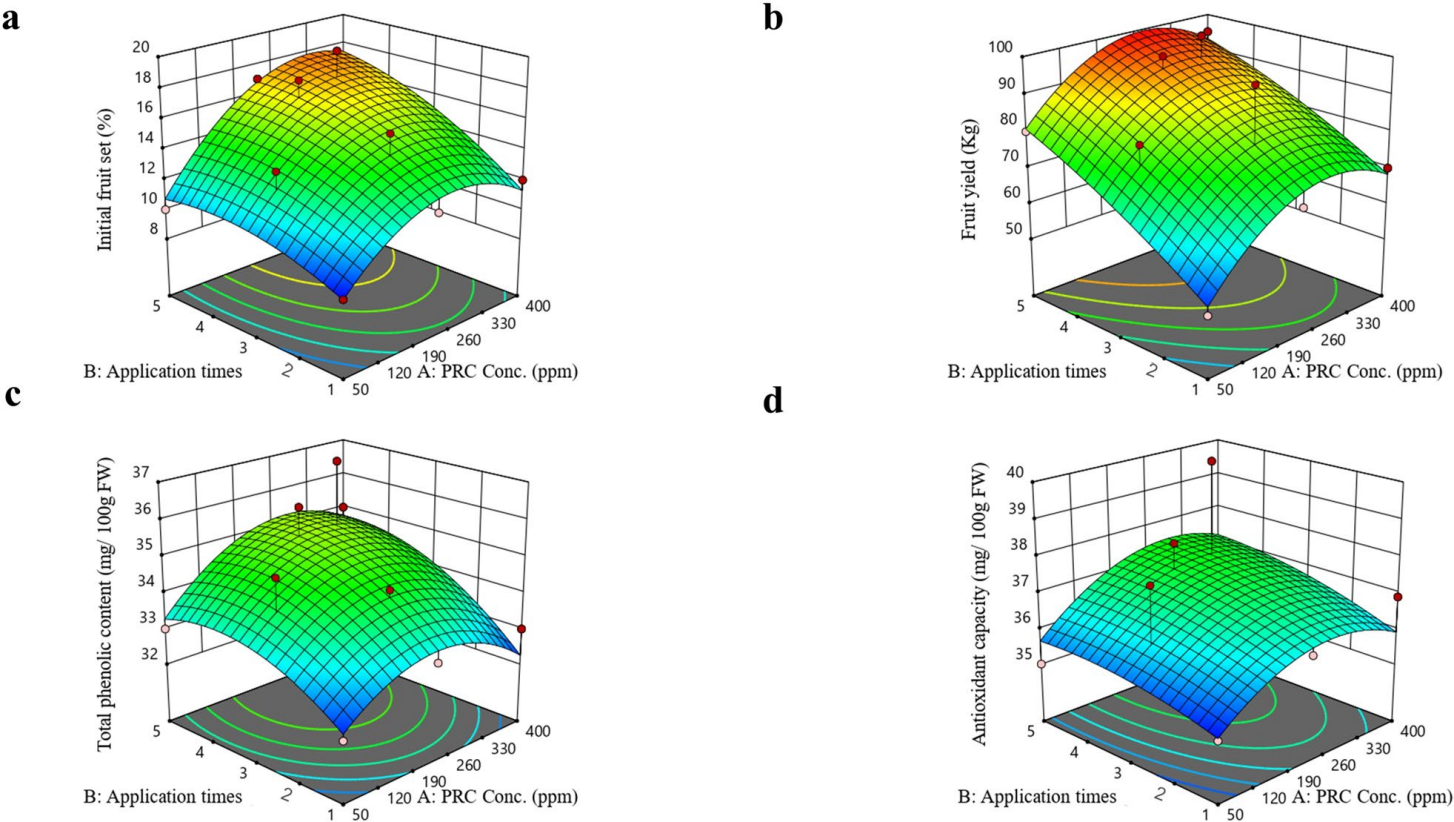
Response surface plots of initial fruit set (a), fruit yield (b), total phenolic content (c), and antioxidant capacity (d) in response to PRC concentration (A), and application times (B). PRC concentrations ranging from 50 to 400 ppm were applied with different timing and repetition schemes as follows: (1) Once, at full bloom stage; (2) Once, 3 weeks after full bloom; (3) Once, 6 weeks after full bloom; (4) Twice, at full bloom stage and 3 weeks after full bloom; (5) Twice, at full bloom stage and 6 weeks after full bloom

To study the correlation between the measured responses and the interactions among the variables under study, 3D surface plots (Fig. 1a-d) were created. By employing the implemented RSM models and derived equations, the optimal conditions for improving productivity, TPC, and TAC of 'Le-Conte' pear were determined. Derived from the results, the optimal PRC concentration treatment fell within the range of 200 to 300 ppm, while the most favorable timing involved two applications (once at the full bloom stage and again three weeks after full bloom). Under these optimized conditions, the experimental values closely matched the predicted values, leading to a notable level of desirability.

### Effect of various PRC treatments on the productivity of 'Le-Conte' pear trees

The results presented in Table 2 present the impact of PRC treatments on pear fruit production. All administered treatments resulted in higher percentages of initial fruit set compared to untreated pear trees. Specifically, the application of PRC at a concentration of 300 ppm resulted in the highest fruit set, reaching 19.07 ± 1.64% and 22.35 ± 1.72% in the first and second seasons, respectively.

**Table 2:** Impact of protocatechuic treatments on initial fruit set, fruit abscission, yield, fruit weight, L/D ratio and specific gravity of 'Le-Conte' pear during 2021 and 2022 seasons

**Treatments**	**Initial fruit set (%)**	**Fruit abscission (%)**	**Yield (Kg/tree)**	**Fruit weight (g)**	**L/D ratio**	**Specific gravity**
**2021 season**						
Control	8.27 ± 1.18^b^	69.07 ± 1.16^a^	40.33 ± 2.45^c^	132.47 ± 4.18^b^	1.01 ± 0.15^b^	1.02 ± 0.03^a^
200 ppm PRC	18.27 ± 1.37^a^	45.74 ± 1.28^b^	80.10 ± 2.73^b^	179.70 ± 8.14^a^	1.20 ± 0.12^a^	1.05 ± 0.02^a^
300 ppm PRC	19.07 ± 1.64^a^	42.60 ± 2.12^c^	100.15 ± 2.43^a^	181.03 ± 7.78^a^	1.26 ± 0.16^a^	1.07 ± 0.02^a^
**2022 season**						
Control	8.60 ± 1.23^b^	67.50 ± 1.12^a^	39.67 ± 1.88^c^	140.93 ± 7.66^b^	1.06 ± 0.14^b^	1.03 ± 0.04^a^
200 ppm PRC	19.77 ± 1.54^a^	42.56 ± 0.77^b^	91.33 ± 2.86^b^	187.21 ± 9.07^a^	1.15 ± 0.17^a^	1.07 ± 0.02^a^
300 ppm PRC	22.35 ± 1.72^a^	38.95 ± 0.89^c^	105.48 ± 1.77^a^	193.70 ± 6.03^a^	1.24 ± 0.13^a^	1.08 ± 0.03^a^

Each value is the mean for five replicates ± standard error. Values followed by different letters differ significantly (*P* ≤ 0.05)

Moreover, the examined treatments significantly decreased fruit abscission compared to the control. Spraying PRC at 300 ppm exhibited the lowest and statistically significant percentages of fruit abscission, measuring 42.60 ± 2.12% and 38.95 ± 0.89% in both investigation seasons, respectively.

In terms of yield, the PRC treatments demonstrated significantly higher yields compared to the untreated trees. Applying PRC at 300 ppm resulted in the highest and statistically significant yields, with 100.15 ± 2.43 Kg/tree and 105.48 ± 1.77 Kg/tree during both seasons, respectively.

Furthermore, all conducted treatments in the current study enhanced fruit weight in both seasons compared with control. Spraying PRC at 300 ppm displayed the highest fruit weight, measuring 181.03 ± 7.78 g and 193.70 ± 6.03 g in the 2021 and 2022 seasons, respectively, while the control group exhibited the lowest fruit weight values.

### The physicochemical characteristics of fruits in response to PRC treatments

The effects of the conducted treatments on the length/diameter ratio, specific gravity, firmness, a* peel, and L* flesh values of 'Le-Conte' pear fruits during the 2021 and 2022 seasons are presented in Tables 2 and 3. The observed differences among the PRC treatments regarding the length/diameter ratio of pear fruits were not statistically significant. However, applying PRC at a concentration of 300 ppm resulted in higher length/diameter ratios compared to the other treatments, recording values of 1.26 ± 0.16 and 1.24 ± 0.13 during the 2021 and 2022 experimental seasons, respectively. No significant variations in the specific gravity of the fruits were observed due to the different applied treatments in 2021 and 2022 seasons. In both seasons, untreated trees exhibited significantly higher fruit firmness compared to the PRC treatments at harvest. The application of PRC at 200 ppm resulted in the lowest firmness values, measuring 67.21 ± 1.61 N and 64.68 ± 0.55 N in the first and second seasons, respectively.

**Table 3:** Impact of protocatechuic treatments on firmness, a* peel, L* flesh, TSS, ascorbic acid and total sugars of 'Le-Conte' pear during 2021 and 2022 seasons

**Treatments**	**Firmness (N)**	a* peel	L* flesh	**TSS (%)**	**Ascorbic acid (mg/100 g FW)**	**Total sugars (%)**
**2021 season**						
Control	77.48 ± 1.24^a^	-23.31 ± 0.77^b^	67.41 ± 1.02^c^	11.82 ± 0.44^c^	6.32 ± 0.41^c^	6.36 ± 0.33^b^
200 ppm PRC	67.21 ± 1.61^c^	-14.07 ± 0.46^a^	72.91 ± 0.91^a^	14.62 ± 0.56^a^	7.09 ± 0.46^a^	8.68 ± 0.24^a^
300 ppm PRC	71.21 ± 1.47^b^	-15.32 ± 0.61^a^	68.76 ± 0.67^b^	13.91 ± 0.76^b^	6.51 ± 0.39^b^	7.43 ± 0.39^b^
**2022 season**						
Control	73.43 ± 0.81^a^	-20.97 ± 0.46^b^	65.37 ± 0.79^c^	12.91 ± 0.62^c^	6.13 ± 0.31^c^	6.42 ± 0.31^c^
200 ppm PRC	64.68 ± 0.55^c^	-12.91 ± 0.49^a^	71.89 ± 0.69^a^	14.93 ± 0.71^a^	6.92 ± 0.28^a^	8.14 ± 0.41^a^
300 ppm PRC	68.43 ± 0.81^b^	-13.99 ± 0.36^a^	68.13 ± 0.68^b^	14.02 ± 0.64^b^	6.37 ± 0.39^b^	7.50 ± 0.37^b^

Each value is the mean for five replicates ± standard error. Values followed by different letters differ significantly (*P* ≤ 0.05)

Concerning the fruit color values of the fruit peel, PRC at 200 ppm exhibited the highest a* peel color values, indicating a lower presence of green color compared to untreated fruits, which exhibited the lowest values. PRC at 200 ppm recorded a* values of -14.07 ± 0.46 and -12.91 ± 0.49 during the 2021 and 2022 seasons, respectively. There were no significant differences observed between PRC treatments. The L* flesh values were significantly affected by the different treatments. PRC at 200 ppm exhibited the highest and statistically significant L* flesh values, measuring 72.91 ± 0.91 and 71.89 ± 0.69 in 2021 and 2022 seasons, respectively.

Table 3 illustrates the influence of different PRC treatments on TSS, ascorbic acid, and total sugars of 'Le-Conte' pear fruits in the 2021 and 2022 seasons. Throughout both seasons, PRC at 200 ppm exhibited significantly higher TSS values compared to the other treatments, measuring 14.62 ± 0.56% and 14.93 ± 0.71% respectively. Also, regarding the total sugar content of the resulting fruits, PRC at 200 ppm displayed the highest total sugar values, measuring 8.68 ± 0.24 and 8.14 ± 0.41% in both seasons, respectively. All PRC treatments successfully preserved the ascorbic acid content of 'Le-Conte' fruits compared to control group. PRC at 200 ppm demonstrated the highest ascorbic acid values, with 7.09 ± 0.46 mg/100 g FW and 6.92 ± 0.28 mg/100 g FW during the 2021 and 2022 seasons, respectively.

### Stone cells and lignin contents

**Fig. 2:**
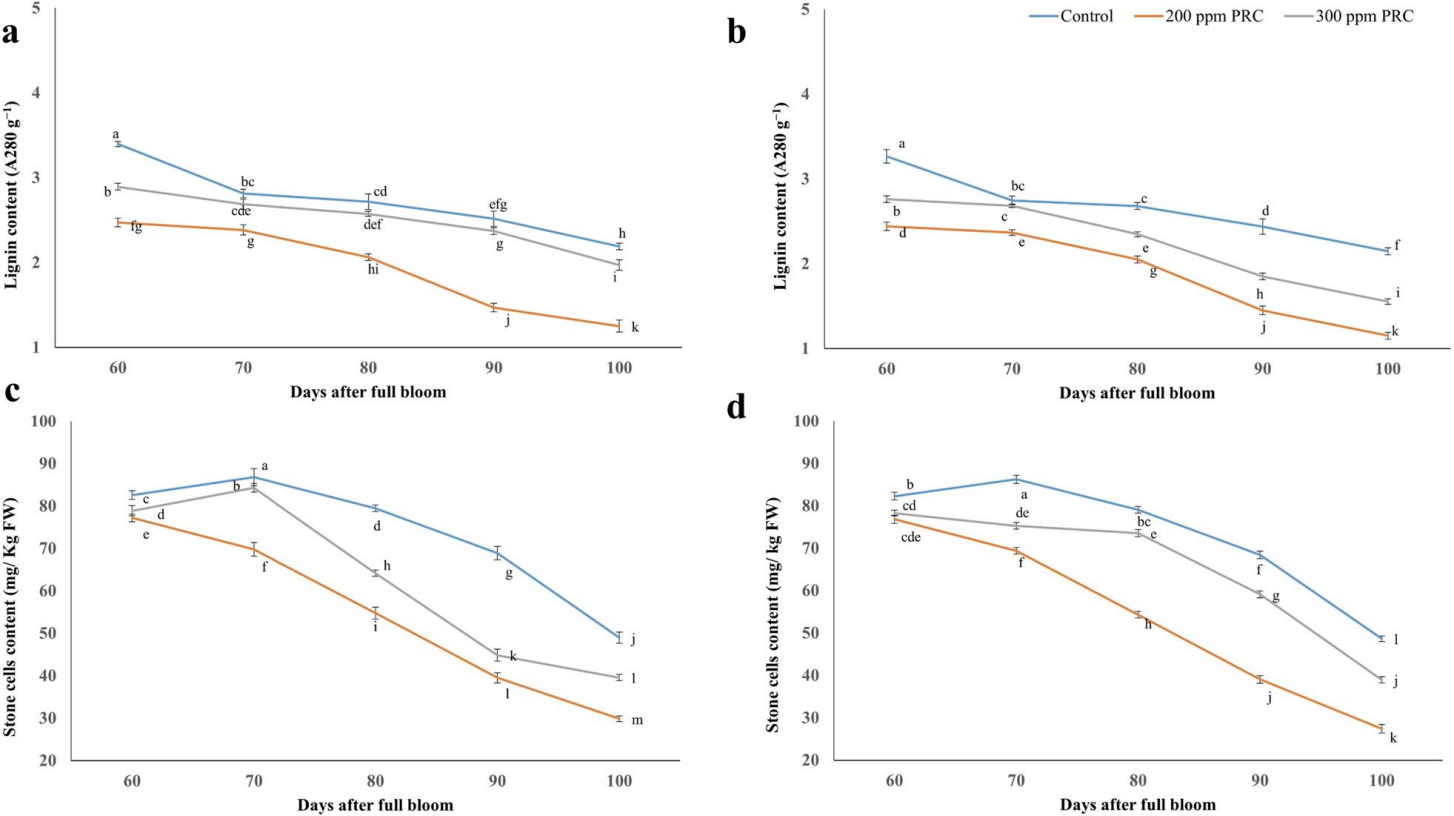
Effect of protocatechuic treatments on lignin and stone cells of 'Le-Conte' pear during 2021 (a, c) and 2022 (b, d) seasons, respectively. Vertical bars indicate the standard error of the means with different letters indicating significant variance (*P* ≤ 0.05) between means, as determined by Duncan's multiple range test

Figure 2 illustrates the impact of PRC treatments on the stone cells and lignin contents of 'Le-Conte' pear fruits at various stages of development during the 2021 and 2022 seasons. During fruit development, the level of lignin displayed a pattern parallel to the change in stone cells content. As the fruits developed, the lignin content decreased. Similar to the stone cells content, the level of lignin remained stably low during the late development stage under all treatments in both seasons. This suggests that the formation of stone cells and lignin synthesis are intricately linked processes, with both exhibiting a decrease during fruit development and reaching a relatively low level in the late stages. Concurrently, the content of stone cells initially increased, followed by a subsequent decrease. This trend was observed in both seasons and across all treatments. In the late development stage, the stone cells content reached a relatively low level, indicating a decline in stone cells formation under all treatments. PRC at 200 ppm demonstrated the lowest stone cells and lignin contents at harvest time in both seasons.

### Total phenolics, and antioxidant capacity content

**Fig. 3:**
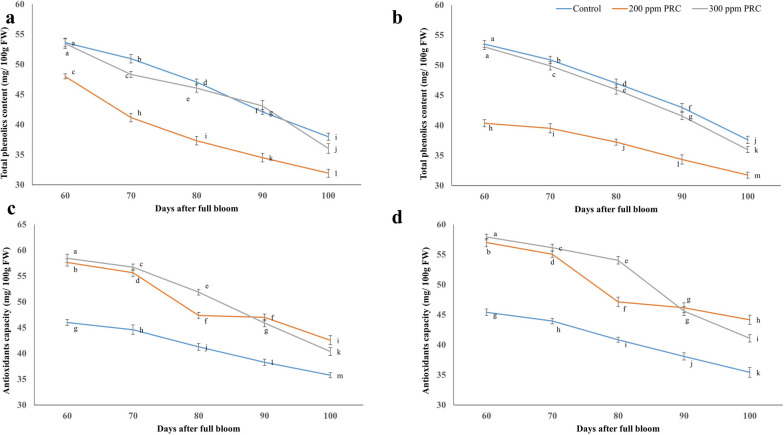
Effect of protocatechuic treatments on total phenolics content, and antioxidant capacity of 'Le-Conte' pear during 2021 (a, c) and 2022 (b, d) seasons, respectively. Vertical bars indicate the standard error of the means with different letters indicating significant variance (*P* ≤ 0.05) between means, as determined by Duncan's multiple range test

Figure 3 illustrates the impact of PRC treatments on the TPC and TAC of 'Le-Conte' pear fruits at various stages of development during the 2021 and 2022 seasons. Significantly higher phenolics levels were observed in the 'Le-Conte' pear fruits treated with PRC compared to the control group, which exhibited the lowest content. The total phenolics content gradually decreased as the fruits advanced in growth. Overall, the trees treated with PRC at a concentration of 200 ppm displayed the lowest and statistically significant TPC compared to the untreated trees (control) throughout both seasons. Furthermore, Fig. 3c, d demonstrates the effect of different PRC concentration treatments on the TAC of 'Le-Conte' pear fruits at various stages of development during the 2021 and 2022 seasons. In this context, the TAC sharply declined during the fruit development stages. The PRC treatments proved to be effective in enhancing the TAC compared to the control group. For instance, the PRC maintained the highest TAC after 100 days of full bloom in the first and second seasons.

### Phenylpropanoid pathway, antioxidants, and quality- related enzymes

The impact of PRC treatments on PAL, C4H, 4CL, and CCR activity is presented in Table 4. All the conducted treatments resulted in lower PAL activity compared to untreated trees. Notably, PRC at 200 ppm exhibited the lowest and statistically significant PAL activity, measuring 182.33 ± 1.88 U/g FW and 170.33 ± 1.97 U/g FW in the first and second seasons, respectively.

**Table 4:** Impact of protocatechuic treatments on phenylalanine ammonium lyase (PAL), cinnamate-4-hydroxylase (C4H), 4-coumarate-CoA Ligase (4CL), and Cinnamoyl-CoA reductase (CCR) of 'Le-Conte' pear during 2021 and 2022 seasons

**Treatments**	**PAL (U/g FW)**	**C4H (U/g FW)**	**4CL (U/g FW)**	**CCR (U/g FW)**
**2021 season**				
Control	205.53 ± 1.59^a^	26.11 ± 0.89^a^	28.82 ± 0.49^a^	118.77 ± 0.68^a^
200 ppm PRC	182.33 ± 1.88^c^	20.78 ± 1.18^c^	22.86 ± 0.41^c^	98.99 ± 0.99^c^
300 ppm PRC	198.63 ± 2.20^b^	22.04 ± 0.95^b^	24.03 ± 0.51^b^	102.65 ± 0.71^b^
**2022 season**				
Control	197.80 ± 1.98^a^	24.36 ± 0.91^a^	25.92 ± 0.31^a^	126.09 ± 0.85^a^
200 ppm PRC	170.33 ± 1.97^b^	19.53 ± 1.44^c^	20.33 ± 0.45^c^	87.90 ± 0.95^b^
300 ppm PRC	179.41 ± 1.98^a^	21.33 ± 0.68^b^	22.37 ± 0.43^b^	98.54 ± 0.63^b^

Each value is the mean for five replicates ± standard error. Values followed by different letters differ significantly (*P* ≤ 0.05)

Significantly higher C4H activity was observed in the control compared to the PRC treatments. Spraying PRC at 200 ppm demonstrated the lowest significant statistical C4H activity, with values of 20.78 ± 1.18 U/g FW and 19.53 ± 1.44 U/g FW in both investigation seasons.

PRC treatments also led to lower 4CL activity compared to untreated trees. Applying PRC at 200 ppm resulted in the lowest significant 4CL activity, measuring 22.86 ± 0.41 U/g FW and 20.33 ± 0.45 U/g FW during the 2021 and 2022 seasons, respectively. Additionally, all the conducted treatments in both seasons decreased CCR activity. Spraying PRC at 200 ppm exhibited the lowest CCR activity, with values of 98.99 ± 0.99 U/g FW and 87.90 ± 0.95 U/g FW compared to the control group, which recorded the highest CCR values in the 2021 and 2022 seasons.

The impact of PRC treatments on CAD activity, Laccase activity, POD activities, and PPO activity at the harvest stage is presented in Table 5. All the conducted treatments resulted in lower CAD activity compared to untreated trees. Notably, PRC at 200 ppm exhibited the lowest and statistically significant CAD activity, measuring 125.36 ± 0.82 U/g FW and 121.10 ± 1.22b U/g FW in the first and second seasons, respectively. The difference in CAD activity between the 200 ppm and 300 ppm PRC treatments was non-significant in the second season.

**Table 5:** Impact of protocatechuic treatments on cinnamyl-alcohol dehydrogenase (CAD), laccase activity, polyphenol oxidase (PPO), and peroxidase (POD) of 'Le-Conte' pear during 2021 and 2022 seasons

**Treatments**	CAD (U/g FW)	Laccase (U/g FW)	PPO (U/g FW)	POD (U/g FW)
**2021 season**				
Control	160.88 ± 0.59^a^	16.23 ± 0.38^c^	1.51 ± 0.19^a^	1.92 ± 0.18^a^
200 ppm PRC	125.36 ± 0.82^c^	25.68 ± 0.37^a^	1.03 ± 0.19^c^	1.45 ± 0.16^c^
300 ppm PRC	136.55 ± 0.42^b^	21.79 ± 0.49^b^	1.17 ± 0.12^b^	1.77 ± 0.19^b^
**2022 season**				
Control	140.02 ± 0.79^a^	20.56 ± 0.57^c^	1.72 ± 0.21^a^	1.88 ± 0.14^a^
200 ppm PRC	121.10 ± 1.22^b^	27.07 ± 0.49^a^	1.08 ± 0.15^b^	1.50 ± 0.03^c^
300 ppm PRC	125.48 ± 0.66^b^	23.85 ± 0.39^b^	1.15 ± 0.20^b^	1.66 ± 0.13^b^

Each value is the mean for five replicates ± standard error. Values followed by different letters differ significantly (*P* ≤ 0.05)

Significantly higher Laccase activity was observed in the tested treatments compared to the control group. Spraying PRC at 200 ppm demonstrated the highest and statistically significant Laccase activity, with values of 25.68 ± 0.37 U/g FW and 27.07 ± 0.49 U/g FW in both investigation seasons, respectively.

Additionally, all the conducted treatments in both seasons reduced fruit PPO activity. Spraying PRC at 200 ppm exhibited the lowest and statistically significant PPO activity, with values of 1.03 ± 0.19 U/g FW and 1.08 ± 0.15 U/g FW compared to the control group, which recorded the highest PPO values in the 2021 and 2022 seasons. The difference in PPO activity between the 200 ppm and 300 ppm PRC treatments was non-significant in the second season. Moreover, PRC treatments led to lower significant POD activity compared to untreated trees. Applying PRC at 200 ppm resulted in the lowest and statistically significant POD activity, measuring 1.45 ± 0.16 U/g FW and 1.50 ± 0.03 U/g FW during the 2021 and 2022 seasons, respectively.

## Discussion

The aim of this study was to examine the impacts of PRC treatments on the productivity and fruit quality of 'Le-Conte' pear. The results demonstrate significant improvements in various aspects of fruit production and quality, validating the efficacy of PRC treatments in enhancing 'Le-Conte' pear cultivation. Based on the results obtained using RSM, this study yielded significant findings regarding treatment concentrations, timing of implementation, and treatment repetition schedules. Based on the findings, the optimal PRC concentration treatment fell within the range of 200 to 300 ppm, while the most favorable timing involved two applications (once at the full bloom stage and again three weeks after full bloom). Under these optimized conditions, the experimental values closely aligned with the predicted values, resulting in a notable level of desirability. The findings reveal that PRC treatments positively influence fruit set percentages, reducing fruit abscission and promoting better fruit retention on the trees. These results are consistent with previous studies on other crops [21, 22], where PRC treatments have shown positive effects on fruit set and abscission rates. The observed effects could be attributed to the role of PRC in promoting hormone balance, enhancing pollination processes, and improving overall fruit development. Additionally, it might be due to its impact on delaying physiochemical alterations that result in the formation of the separation zone between fruit and shoots. The abscission zone is believed to be formed through enzymatic activity that breaks down cell wall components such as pectin, cellulosic materials, and non-cellulosic polysaccharides. Migration of calcium and magnesium from the cell walls occurs in that section leading to abscission [39]. Furthermore, the application of PRC treatments results in increased fruit yield. This can be attributed to the constructive impact of PRC on the fruit set observed in this study. Where PRC treatments have shown significant yield enhancement effects. The improved yield can be attributed to the positive influence of PRC on floral development, fertilization, and fruit development.

Regarding fruit quality, PRC treatments led to enhancements in multiple physicochemical characteristics. The enhanced color in PRC-treated fruits can be attributed to improved color values, including lower a* values and higher flesh lightness. These improvements are a result of PRC's antioxidant properties, which help delay undesirable color changes and slow down fruit senescence [22].

Moreover, the influence of PRC treatments on the accumulation of sugars and ascorbic acid content aligns with the findings of previous studies [40, 41]. PRC has been reported to control carbohydrate metabolism and enhance sugar content in fruits [40]. Additionally, the antioxidant properties of PRC can protect ascorbic acid from degradation and maintain its content in fruits [41].

 According to Lu et al. [8], plant growth promoters play a pivotal role in controlling and regulating biological processes. Researchers argue that these regulators enhance the mobility of plant fluids, thereby facilitating nutrient transfer in the phloem. Furthermore, they may influence sugar transport from the phloem, and plant growth regulators can also impact metabolism and the arrangement of sugars and their metabolites [8]. According to Zheng et al. [42], the decrease in ascorbic acid levels is primarily attributed to the oxidation of dehydroascorbic acid to diketogulonic acid, and this oxidation process is facilitated by the enzyme ascorbate oxidase. This suggests that PRC plays a role in preserving ascorbic acid content. Furthermore, PRC treatments had a significant effect on the phenolic profile and TAC of 'Le-Conte' pears. PRC at 200 ppm recorded balanced concentrations of TPC and TAC which can be attributed to the upregulation of phenylpropanoid biosynthesis pathways during fruit development [19]. Additionally, the activity of key enzymes involved in phenylpropanoid metabolism, such as PAL, C4H, 4CL, and CCR, as observed in this study, plays a pivotal role in the accumulation of phenolic compounds and TAC [19].

Researchers have focused on the process of stone cells development and buildup in pears, which leads to lower interior fruit quality [43]. Despite accounting for just 20-30% of mature stone cells [16], lignin has been postulated to play an important function in stone cells growth. As a result, we inferred that the decrease in stone cells content during the late growth stage might be related to pear fruit cell elongation, which collects sugar and other organic substances in accordance with Yan et al. [12]. Lignin, a complex natural polymer, is synthesized through the phenylpropanoid pathway, with the initial step catalyzed by cinnamoyl-CoA reductase [44–46]. PAL is considered a crucial enzyme in the phenylpropanoid pathway, and alterations in its activity are indicative of the level of plant-induced resistance [47]. Changes in TPC are primarily associated with the activities of C4H and PPO. C4H, which acts downstream of PAL, promotes synthesis [48], while PPO is responsible for converting phenols into quinones [49], which are further transformed into melanin pigment through non-quinone oxidation-reduction reactions [50]. Modifications in lignin content are influenced by changes in the activities of upstream enzymes [4]. Consequently, elicitor-induced alterations in lignin content may impact the activities of key rate-limiting enzymes involved in the conversion of upstream products into lignin. Lignin synthesis typically involves the following steps; First, 4CL converts 4-coumaric acid, generated by the phenylpropanoid pathway, into 4-coumaroyl-CoA. Second, CCR catalyzes the production of 4-coumaraldehyde, which serves as the direct precursor for lignin biosynthesis, and is further transformed by CAD [4]. Also, 4CL is considered a key enzyme in the lignin metabolism pathway [51], it was notably inhibited in the treated trees compared to the control group. This decrease ensures the lower conversion of phenylpropanoid pathway products into substrates for the lignin biosynthetic pathway [14]. Additionally, PRC treatments led to a significant decrement in the activities of CCR and CAD, which function as rate-limiting enzymes in the final stages of lignin biosynthesis, facilitating the conversion of ferulic acid CoA into cinnamaldehyde and playing a significant role in lignin content regulation [52].

The fluctuating pattern of stone cells content during fruit development, as observed in this study, suggests a potential relationship between stone cells formation and the activities of enzymes involved in lignin biosynthesis. One such enzyme is PAL, which catalyzes the conversion of phenylalanine to cinnamic acid, a precursor for lignin synthesis [14]. The lower PAL activity observed in the PRC-treated groups compared to the control suggests an upregulation of the lignin biosynthesis pathway, potentially contributing to the development of stone cells [13]. Additionally, the activities of enzymes such as C4H, 4CL, and CCR are also crucial for lignin synthesis [18]. The lower activities of these enzymes observed in the PRC-treated groups, in comparison to the control, further support the idea of inhibited lignin biosynthesis. The reduction in these enzyme activities may lead to decreased lignin deposition, potentially contributing to suppressing the development and accumulation of stone cells in 'Le-Conte' pears.

Moreover, the activities of enzymes such as CAD, POD, and PPO participate in lignin polymerization and oxidation processes [8, 18]. The lower activities of these enzymes observed in the PRC-treated groups compared to the control suggest an inhibition of lignin polymerization and oxidation, which may contribute to the formation and accumulation of stone cells in accordance with Shi et al. [4]. Taken together, the observed relationship between stone cells content and the activities of enzymes involved in lignin biosynthesis and metabolism suggests that PRC treatments may influence the deposition and composition of lignin, thereby affecting the formation and characteristics of stone cells in 'Le-Conte' pears. Furthermore, laccase remained considerably higher in the treatment group than in the control, indicating their catalytic role in lignin synthesis and accumulation [53, 54]. Lignin is catalyzed mainly by laccase; previous research has demonstrated that exogenous substance treatments can control lignin content [55]. In our study, PRC treatments effectively decreased the activities of lignin biosynthesis-related enzymes compared to the control, resulting in regressed lignin accumulation (*P* < 0.05).

Overall, the findings of this study suggest that PRC treatment at 200 ppm is more recommended than 300 ppm. This approach serves as an effective strategy for achieving a balance between enhancing the productivity and fruit quality of 'Le-Conte' pears. The positive effects of PRC treatments on fruit set, fruit retention, yield, physicochemical characteristics, sugar accumulation, ascorbic acid content, phenolic profile, and TAC, and inhibition in lignin and stone cells accumulation highlight its potential for commercial application.

## Conclusion

In conclusion, the results of this study highlight the significant positive effects of PRC treatments on the productivity and fruit quality of 'Le-Conte' pears. PRC treatments enhance fruit set percentages, reduce fruit abscission, and increase fruit yield. The treated fruits also exhibit improved physicochemical characteristics, including enhanced color with moderate firmness values. Moreover, PRC treatments positively influence the accumulation of sugars, ascorbic acid content, and TAC. Additionally, PRC treatments modulate the activity of key enzymes involved in phenylpropanoid metabolism, such as PAL, C4H, 4CL, CCR, CAD, in addition to other related enzymes; POD, laccase, and PPO. These findings highlight the potential of PRC at 200 and 300 ppm, applied twice (once at the full bloom stage and again three weeks after full bloom) treatments as a comprehensive approach for enhancing yield, improving fruit quality, and influencing the enzymatic processes related to phenylpropanoid metabolism in 'Le-Conte' pears. Overall, the findings of this study suggest that PRC treatment at 200 ppm is highly recommended. This approach serves as an effective strategy for achieving a balance between enhancing the productivity and fruit quality of 'Le-Conte' pears. Future research endeavors should focus on optimizing PRC treatment protocols and unraveling the underlying mechanisms, particularly in cultivars characterized by higher stone cells content, to facilitate practical implementation.

### Supplementary Information

**Additional file 1: Table S1.** Main physical and chemical properties of the experimental field soil. **Table S2.** Main properties of applied irrigation water.

## Data Availability

The original contributions presented in the study are included in the article, and further inquiries can be directed to the corresponding author.
