# Supplementary material for: Enhancing productivity, modifying biochemical parameters, and regulating the phenylpropanoid pathway in 'Le-Conte' pears through optimal protocatechuic acid treatments
Source: BMC Plant Biol. 2024 Jan 15;24:50. doi: 10.1186/s12870-023-04715-9 (PMC10789004; doi:10.1186/s12870-023-04715-9)
Supplement: Supplementary file 1 — Additional file 1: Table S1. Main physical and chemical properties of the experimental field soil. Table S2. Main properties of applied irrigation water. [file 12870_2023_4715_MOESM1_ESM.pdf]

Table S1. Main physical and chemical properties of the experimental field soil.

Clay (%)	Silt (%)	Sand (%)	Soil texture	pH	EC (dS/m)	Ca (meq/L)	OM (%)
5.4	3.7	90.9	Sand	7.91	1.03	11.2	0.1

Table S2. Main properties of applied irrigation water.

pH	EC (dS/m)	HCO_3 (meq/L)	Cl (meq/L)	SO_4 (meq/L)	Na (meq/L)	K (meq/L)	Ca (meq/L)	Mg (meq/L)
7.2	3.8	3.65	26.66	10.23	35.02	0.51	7.88	6.12
